# Transcriptome and Metabolome Analyses Reveal Differences in the Accumulation of Key Metabolites in Various Tissues of *Lonicera macranthoides*

**DOI:** 10.3390/metabo16010005

**Published:** 2025-12-22

**Authors:** Zhengchun Li, Zijing Zhou, Ninghong Qiu, Fengfei Yang, Hua Feng, Tangyan Li, Qiandong Hou

**Affiliations:** 1 Key Laboratory of Conservation, Development and Utilization of Karst Characteristic Biological Resources, Department of Modern Agriculture, Zunyi Vocational and Technical College, Zunyi 563006, China; zcliah@163.com (Z.L.);; 2 Institute for Forest Resources & Environment of Guizhou/College of Forestry, Guizhou University, Guiyang 550025, China; 3 School of Ethnic-Minority Medicine, Guizhou Minzu University, Guiyang 550025, China

**Keywords:** *Lonicera macranthoides*, transcriptome, metabolome, hederagenin-based saponins, flavonoid biosynthesis

## Abstract

Background: *Lonicera macranthoides* is a valuable traditional Chinese medicinal plant, enriched in secondary metabolites that exert significant therapeutic effects against specific diseases. However, the differences in the accumulation of key metabolites across various tissues of this plant remain poorly understood. Methods: This study analyzed the transcriptomes and metabolomes of three key tissues (flowers, leaves, and fruits) in *Lonicera macranthoides*. Results: Transcriptome sequencing identified 7321 differentially expressed genes (DEGs) common to flowers, fruits, and leaves. Among the top 20 KEGG pathways enriched by these DEGs, metabolic pathways and biosynthesis of secondary metabolites were prominently represented. At least 70 *CYP* genes and 12 *UGT* genes were differentially expressed, with a greater proportion of these genes being up-regulated in flowers or fruits. DEGs involved in flavonoid biosynthesis include *CHI*, *CHS*, and *FLS*, with most of these genes being up-regulated in fruits. Metabolomics analysis identified 4961 metabolites across the three tissues. KEGG pathway classification of these DAMs showed that a large proportion are involved in metabolic pathways and secondary metabolite biosynthesis. Conjoint analysis of the transcriptomic and metabolomic data revealed that the most significantly enriched were metabolic pathways and biosynthesis of secondary metabolites. Integrative analysis of DEGs and DAMs indicated that flowers and fruits are likely key tissues for the biosynthesis of hederagenin-based saponins, while fruits may serve as critical organs for flavonoid biosynthesis. Conclusions: This study provides novel insights into the tissue-specific differential accumulation of metabolites in *Lonicera macranthoides*, and lays a crucial foundation for further investigating the underlying molecular regulatory mechanisms.

## 1. Introduction

“Shan Yin Hua” (*Lonicera macranthoides*), also known as honeysuckle, is a medicinal plant belonging to the genus Lonicera within the *Caprifoliaceae* family [1]. It possesses antiviral properties and other health benefits, and is widely cultivated in China [1]. As a traditional Chinese medicine, *Lonicera macranthoides* contains a wealth of secondary metabolites beneficial to human health, making it widely used as a functional food [1]. Due to its vibrant appearance and distinctive floral fragrance, it is also cultivated as an ornamental plant. One of the primary metabolites of *Lonicera macranthoides* is hederagenin-based saponins, such as macranthoidin A, macranthoidin B, and dipsacoside B, which have been demonstrated to possess significant antiviral activity [2,3]. Research demonstrated that *Lonicera macranthoides* contains approximately 2000 fold more hederagenin-based saponins than the common honeysuckle (*Lonicera japonica*) [3]. Additionally, *Lonicera macranthoides* is rich in antioxidant flavonoids and the antibacterial compound chlorogenic acid [1,4,5]. In actual production, the blooming flower are the primary medicinal part harvested. Nevertheless, the ripe leaves and fruits also contain substantial amounts of flavonoids, hederagenin-based saponins, chlorogenic acid, and other bioactive metabolites [3]. Therefore, investigating the differences in metabolite accumulation among the flowers, leaves, and fruits of *Lonicera macranthoides* carries significant scientific importance for the in-depth development and utilization of its medicinal potential.

The biosynthesis pathway of hederagenin-based saponins in *Lonicera macranthoides* involves the processes of initiation, triterpene skeleton construction, and glycosylation modification [3]. During the biosynthesis process, squalene cyclooxygenase, oleanolic acid synthase, and β-amyrin synthase are crucial enzymes for the construction of hederagenin-based saponins [6,7]. In *Barbarea vulgaris*, eight cytochrome P450 (CYP) 72A genes and QTLs (quantitative trait locus) for saponin accumulation were co-localized, with CYP72A552 oxidizing oleanolic acid to hederagenin at the C-23 position [8]. In the process of metabolite processing and modification, CYPs and UDP-glucosyltransferases (UGTs) play a crucial role. Research indicated that genes associated with the biosynthesis of hederagenin-based saponins in *Lonicera macranthoides* exhibit higher expression levels than in common honeysuckle, such as oleanolic acid synthase (OAS) and members of the *UGT73* family [3]. Triterpenoid saponins occur naturally in certain plants, and UGTs are key enzymes in the modification process of triterpenoid compounds [9]. LmUGT73P1 catalyzes the conversion of cauloside A into α-hederin, while LmOAS1 efficiently catalyzes the C-28 oxidation of β-amyrin [3]. Interestingly, genome sequencing results indicate that *LmOAS1* and *LmUGT73P1* exhibit neighborhood replication within the genome [3].

*Lonicera macranthoides* is also enriched abundant flavonoid metabolites. Flavonoids not only play crucial roles in plant growth, development, and stress resistance, but also function as active components with significant effects in antioxidant defense and disease treatment [10]. The biosynthesis for flavonoids in plants is the phenylpropanoid pathway. Phenylalanine is converted into p-coumaroyl-CoA through the catalysis of phenylalanine ammonia lyase, cinnamic acid 4-hydroxylase, and 4-coumarate:CoA ligase [11,12]. Subsequently, specific flavonoid compounds are formed through the action of chalcone synthase (CHS), chalcone isomerase (CHI), and various CYP enzymes [11]. In recent years, exploring differences in plant flavonoid synthesis through transcriptomic and metabolomic sequencing has become an effective research approach.

Previous studies have investigated key genes in the hederagenin-based saponin biosynthesis pathway of *Lonicera macranthoides* [3], but differences in tissue-specific synthesis have not yet been reported. In *Lonicera macranthoides*, flowers, fruits, and leaves serve as key sources for extracting flavonoid and saponin, yet the differences in metabolite levels among these tissues remain unclear. Furthermore, whether genes participating in the biosynthesis of key metabolites exhibit tissue-specific expression patterns in *Lonicera macranthoides* remains to be elucidated. Advances in sequencing technology have pointed the way toward resolving these scientific questions, with reports already utilizing integrated transcriptomic and metabolomic analyses to identify genes and pathways associated with key metabolites. Advances in sequencing technology have pointed the way toward resolving these scientific questions. However, no studies have yet investigated the differences in metabolite accumulation across various tissues of *Lonicera macranthoides* using metabolomics and transcriptomics approaches, particularly in leaves and fruits. For instance, integrated analysis of transcriptomic and metabolomic data has identified key regulatory genes involved in the efficient production of flavonoids and styrene compounds using grape rootstock tissue [13]. Consequently, this study aims to investigate differences in the accumulation levels of key metabolites (saponins or flavonoids) in various tissues of *Lonicera macranthoides*. In this research, we collected flowers, fruits, and leaves of *Lonicera macranthoides* for transcriptome sequencing and metabolome profiling. By comparing differences in the accumulation of saponin or flavonoid metabolites and key gene expression patterns across these tissues, this study provides a theoretical foundation for the efficient extraction of valuable bioactive compounds from various tissues of *Lonicera macranthoides*, offering new insights into the effective utilization of biological resources.

## 2. Materials and Methods

### 2.1. Plant Materials

**Figure 1:**
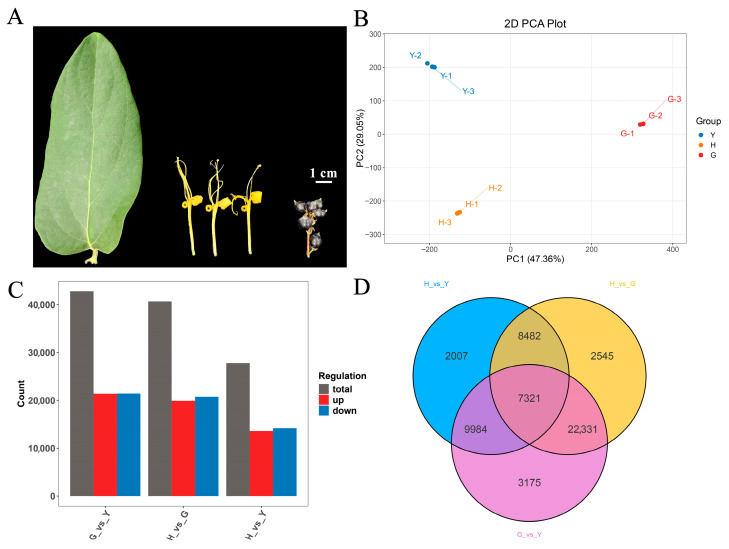
Phenotypic analysis and transcriptomic sequencing of leaves, flowers, and fruits of *Lonicera macranthoides*. (**A**) Phenotypic characteristics of leaves, flowers, and fruits of *Lonicera macranthoides*, with a scale bar of 1 cm. (**B**) Transcriptome PCA. (**C**) Analysis of DEGs across different tissues. (**D**) Venn diagram of DEGs in the three tissues.

The experimental materials were ripened leaves (leaf age is 165–200 days), blooming flowers (golden yellow, the petals are perpendicular to the stamens, and petal curling), and ripened fruits (55 days after flowering, purple-black) of *Lonicera macranthoides*, with sample appearance shown in Figure 1A. The plants were 10 years old, with three replicate samples collected from each of three plants originating from the same clonal line. Plants were cultivated at the *Lonicera macranthoides* Germplasm Resource Nursery in Zhengchang Town, Suiyang County, Zunyi City, Guizhou Province, China (27.89° N, 107.25° E, elevation 826 m). The planting pattern was 2 m between plants and 2.5 m between rows. The experimental materials were collected in November 2024. All biological replicates were maintained at the same developmental stage. After collection, samples were rapidly frozen using liquid nitrogen and stored at −80 °C for future use. All samples were collected with three biological replicates.

### 2.2. Transcriptome Sequencing and Analysis

Total RNA was extracted from nine collected samples (three tissues × three replicates) using an RNA extracted kit (Tiangen, Beijing, China). RNA concentration and integrity were assessed using a Qubit 4.0 Fluorometer/MD Microplate Reader (Thermo Fisher, Waltham, MA, USA) and a Qsep400 Bioanalyzer (BIOptic, Changzhou, China). Qualified RNA was converted to cDNA using the Clontech SMARTer PCR cDNA Kit (Takara, Dalian, China). After terminal repair and purification, PCR amplification was carried out to construct the final cDNA library. The insert size was determined using a fragment analyzer, and paired-end sequencing was performed on the Illumina platform. Strict quality control of data was performed using FASTP 1.0 software [14]. Clean reads were assembled using Trinity [15], and the integrity of the assembled transcripts was evaluated using the Busco v5.6.1 software [16].

### 2.3. Differential Gene Expression (DEGs) Analysis

Using the RSEM and bowtie2 tool, transcript or gene expression levels are measured via FPKM (Fragments Per Kilobase of transcript per Million fragments mapped) [17,18]. Differential expression analysis was performed between sample groups using the DESeq2 package [19]. The Benjamini–Hochberg method was applied to correct *p*-values for multiple hypothesis testing, yielding the False Discovery Rate (FDR). Differentially expressed genes were selected based on the criteria |log_2_Fold Change| ≥ 1 and FDR < 0.05. These genes were annotated and classified using the Kyoto Encyclopedia of Genes and Genomes (KEGG) and Gene Ontology (GO) databases.

### 2.4. Quantitative Real-Time PCR (RT-qPCR) Analysis

RNA was extracted from flowers, leaves, and fruits. The first strand of cDNA was synthesized using the PrimeScript RT Reagent Kit with gDNA Eraser (TaKaRa, Dalian, China). Referencing differentially expressed genes from RNA-seq, Primer Premier 5.0 (PREMIER Biosoft, San Francisco, CA, USA) software was used to design RT-qPCR specific primers for these genes (Table S1). A total of 15 differentially expressed genes were selected for RT-qPCR validation. RT-qPCR experiments were performed using the CFX96 Real-Time PCR System (Bio-Rad, Hercules, CA, USA) with 2× RealStar Fast SYBR qPCR Mix (GenStar, Beijing, China). PCR reaction system contained 1 µL of cDNA template, 5 µL of Mix, 0.3 µL of forward primer (10 µM), 0.3 µL of reverse primer (10 µM), and 3.4 µL of ddH_2_O. The *18S rRNA* was used as the internal reference gene. The relative expression levels of genes were calculated using the 2^−∆∆CT^ method [20]. For each sample, analyses were performed with three biological and three technical replicates.

### 2.5. Metabolite Extraction, Detection, and Analysis

The collected samples were vacuum freeze-dried for 63 h and subsequently ground into a fine powder. Take 50 mg of powder and add 1200 μL of 70% methanol aqueous internal standard extraction solution pre-cooled to 20 °C. The mixture was vortexed for 30 s every 30 min, with this extraction process repeated 6 times. The samples were centrifuged at 12,000 rpm for three minutes, and the supernatant was collected. Subsequently, the supernatant was filtered thought a 0.22 μm pore size microporous membrane, and the filtrate was transferred to injection vials for storage prior to UPLC-MS/MS analysis. Subsequently, the supernatant was filtered through a 0.22 μm pore size microporous membrane, and the filtrate was transferred to injection vials for storage prior to UPLC-MS/MS analysis.

Substances were separated using an LC-30A ultra-high-performance liquid chromatograph (Shimadzu, Tokyo, Japan) with the following column conditions: Waters ACQUITY UPLC HSS T3 Column 1.8 µm, 2.1 mm × 100 mm. Mobile phase A was ultrapure water (0.1% formic acid). Mobile phase B was acetonitrile (0.1% formic acid). The column temperature was maintained at 40 °C. The flow rate was 0.40 mL/min. The injection volume was 4 µL. The mobile phase gradient conditions for the T3 column are shown in Table S2. The mass spectrometry conditions for the AB TripleTOF 6600 (Foster City, CA, USA) are shown in Table S3.

Raw data were converted to mzML format using ProteoWizard, with peak extraction performed via the XCMS program. Peaks with a missing rate > 50% were filtered out, and blank values were imputed using KNN + 1/5 minimum value. Peak area correction was applied using the SVR method. Metabolite identification was conducted by searching the laboratory’s own database, public databases, prediction databases, and the metDNA method. Finally, compounds with a composite identification score ≥ 0.5 and QC samples with CV < 0.5 were extracted. Positive and negative modes were merged, retaining only the compound with the highest confidence level and lowest CV. Metabolites with VIP (Variable Importance in Projection) > 1, as well as those with fold change ≥ 2 and fold change ≤ 0.5, were considered significantly different.

### 2.6. Correlation Analysis Between Metabolome and Transcriptome

Correlation analysis was performed using the quantitative values of genes and metabolites across all samples. The correlation method employed the cor function in R to calculate Pearson correlation coefficients between genes and metabolites. To ensure the statistical reliability and biological significance of the association analysis, we filtered the fold change values of relevant genes and metabolites based on the correlation results with an absolute correlation coefficient (|r|) > 0.8 and a *p*-value < 0.05.

### 2.7. Statistical Analysis

Results are presented as the mean ± standard deviation (SD) of three independent replicate data points. Differences were calculated using one-way analysis of variance (ANOVA) and Duncan’s post hoc test in SPSS 26 software (SPSS Inc., Chicago, IL, USA), with *p* < 0.05 set as the threshold for significant differences. Results were visualized using Origin 2021 (OriginLab, Northampton, MA, USA).

## 3. Results

### 3.1. Phenotypes and RNA-Seq of Lonicera macranthoides

*Lonicera macranthoides* is a traditional Chinese medicinal herb with extensive cultivation, serving as a vital raw material for extracting multiple medicinal components. I In practical production, the flowers serve as the primary source for extracting these bioactive medicinal constituents. Notably, the leaves and fruits of *Lonicera macranthoides* also accumulate substantial amounts of hederagenin-type saponins and flavonoids. To investigate differences in metabolite accumulation, we collected blooming flowers, ripe leaves, and ripe fruits from *Lonicera macranthoides*, establishing three independent biological replicates for each tissue (Figure 1A). Transcriptome and metabolome profiling were subsequently performed, followed by integrated analysis.

Sequencing performed on the Illumina platform yielded 80.87 Gb of raw data. Following quality control, each sample achieved over 7 Gb of clean data. Clean reads ranged from 93.52% to 95.83%, with Q30 scores exceeding 97% (Table S4). Assembly yielded a total of 127,572 Unigenes (Table S5). Additionally, these Unigenes were aligned against various databases (Table S6). Using the BUSCO v5.6.1 software to evaluate transcriptome assembly results, it was found that Complete BUSCOs (C) accounted for 98.5% of the assembly, demonstrating high assembly completeness (Figure S1A). Among the 98.5% of BUSCO genes, 16.9% were single-copy genes, while 81.6% were duplicated genes.

### 3.2. DEG Identification and Analysis

**Figure 2:**
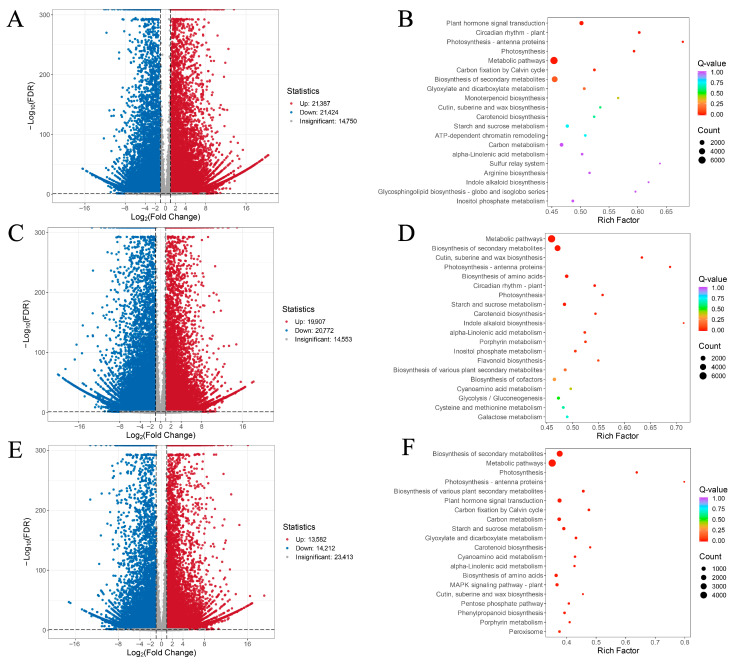
Volcano plots and KEGG pathway analysis of transcriptomic DEGs. (**A**,**C**,**E**) Volcano plots of G vs. Y, H vs. G, and H vs. Y DEGs, where blue indicates down-regulated genes and red indicates up-regulated genes. (**B**,**D**,**F**) KEGG pathway analysis of G vs. Y, H vs. G, and H vs. Y DEGs.

Principal component analysis (PCA) of leaves, flowers, and fruits revealed distinct sample clusters, indicating that biological replicates and sequencing data are suitable for subsequent analyses (Figure 1B). Based on DEG screening criteria, 42,811 DEGs were identified between G and Y (G vs. Y), comprising 21,387 upregulated and 21,424 downregulated genes; between H and G (H vs. G), 40,679 DEGs were identified, comprising 19,907 upregulated and 20,772 downregulated genes; between H and Y (H vs. Y), 27,794 DEGs were identified, comprising 13,582 upregulated and 14,212 downregulated genes (Table S7, Figure 1C). The relatively high number of differentially expressed genes may be attributed to the large gene content within the *Lonicera macranthoides* genome and the pronounced tissue-specific expression patterns [3]. Joint analysis of the three comparison groups identified 7321 differentially expressed genes (DEGs) shared among the H, G, and Y groups (Figure 1D). Volcano plots were further visualized for G vs. Y, H vs. G, and H vs. Y (Figure 2A,C,E).

GO enrichment analysis of DEGs revealed that they could be primarily categorized into biological process (BP), cellular component (CC), and molecular function (MF). Among these, cellular processes, metabolic processes, and catalytic activities showed higher enrichment of DEGs (Figure S1B–D). Further analysis of the top 20 KEGG pathways enriched by DEGs across the three comparison groups (G vs. Y, H vs. G, and H vs. Y) revealed several significant pathways, including metabolic pathways, biosynthesis of secondary metabolites, photosynthesis-related pathways, plant hormone signal transduction, and starch and sucrose metabolism. (Figure 2B,D,F). Among the identified transcription factors, the three most abundant families are C2H2, bHLH, and AP2/ERF (Figure S2).

**Figure 3:**
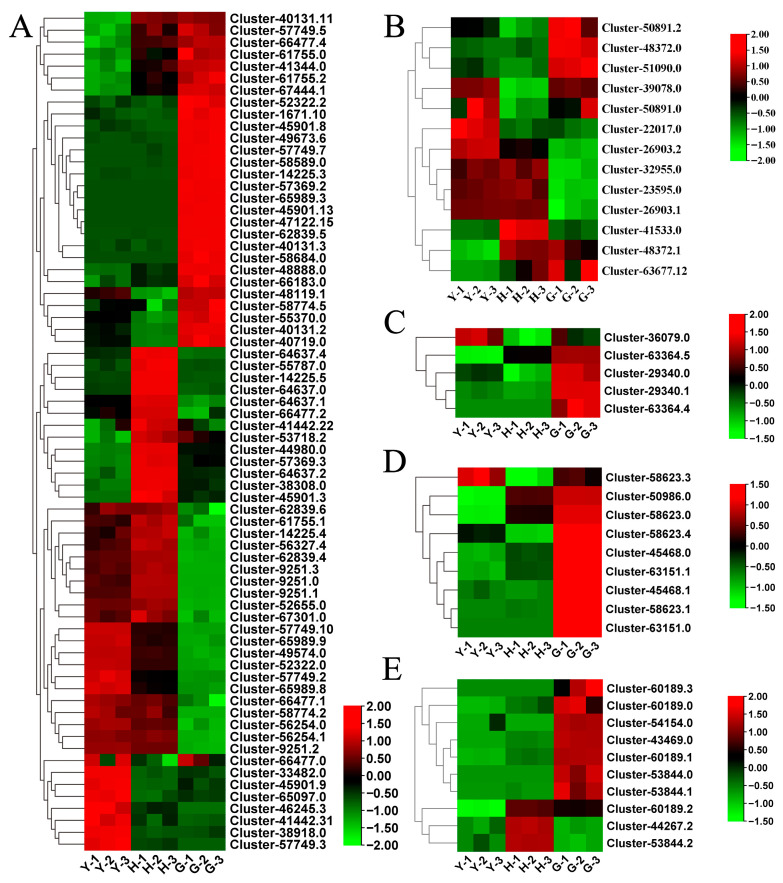
Differential expression of genes related to hederagenin-based saponins and flavonoid biosynthesis in leaves, flowers, and fruits of *Lonicera macranthoides*. (**A**,**B**) Differential expression of *CYP* and *UGT* genes, respectively. (**C**–**E**) DEGs of *CHI*, *CHS*, and *FLS*, respectively.

Previous studies have identified CYP and UGT enzymes as participants in the biosynthesis of hederagenin-based saponins. Among the DEGs in this study, we detected 70 relatively differentially expressed *CYP* genes. Total of 23 *CYP* genes were significantly upregulated in fruit, 42 in flowers, and 29 in leaves (Figure 3A). Based on previous studies, the gene involved in saponin synthesis is CYP72. In this study, four CYP72 homologs were identified: Cluster-1671.10, Cluster-41442.22, Cluster-41442.31, and Cluster-58684.0. Additionally, 12 differentially expressed *UGTs* were identified, with the highest number (7) upregulated in fruit, while 5 each were upregulated in leaves and flowers (Figure 3B). Among these, the homologous gene involved in saponin synthesis may be Cluster-41533.0. These results suggest that *Lonicera macranthoides* possesses the capacity to synthesize hederagenin-based saponins in its flowers, fruit, and leaves.

Research has demonstrated that the flowers of *Lonicera macranthoides* are enriched with abundant flavonoids. In this study, we identified several key genes in flavonoid biosynthesis pathways that exhibit differential expression across different tissues. Among these, five *CHI* genes were predominantly upregulated in fruit (Figure 3C). Nine *CHS* genes were identified, also showing significant upregulation in fruit (Figure 3D). Ten *FLS* genes were identified, with most exhibiting differential expression in fruit (Figure 3E). These results suggest that *Lonicera macranthoides* fruit may accumulate significant levels of flavonoid compounds.

### 3.3. qRT–PCR Verification of DEGs

**Figure 4:**
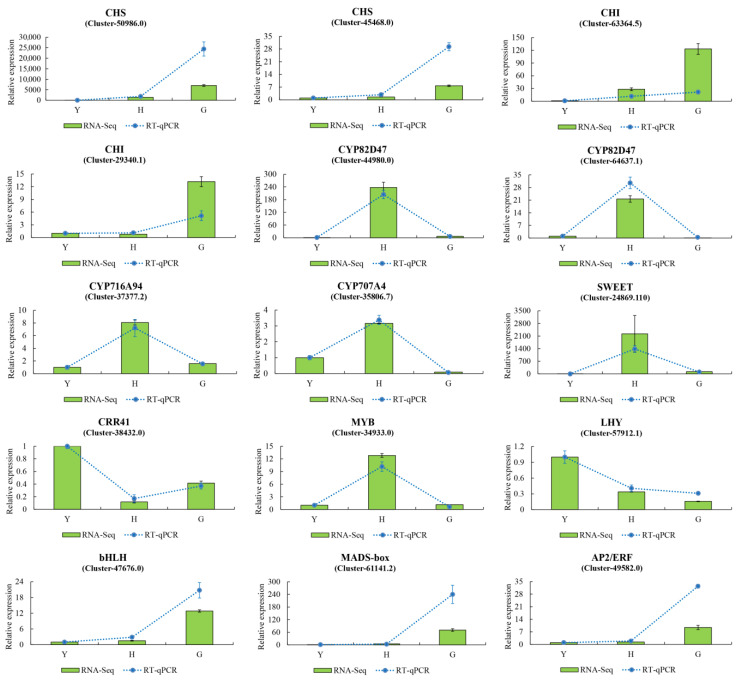
RT-qPCR validation of DEGs in the transcriptome. Results are presented as the mean ± standard deviation (SD) of three replicates. Green bars represent transcriptome results, while blue dashed lines indicate RT-qPCR results.

Based on the distribution of DEGs in enriched KEGG pathways, 15 genes were randomly selected for RT-qPCR validation (Figure 4). These genes encompassed hederagenin-based saponin biosynthesis, flavonoid metabolism, carbohydrate metabolism, and several transcription factors. Experimental data demonstrated that the relative expression levels of genes determined by RT-qPCR were consistent with the expression trends of FPKM values derived from RNA-seq analysis. These RT-qPCR results thus validated the reliability and reproducibility of the transcriptome sequencing data. 

### 3.4. Metabolomics Analysis of Different Tissues of Lonicera macranthoides

**Figure 5:**
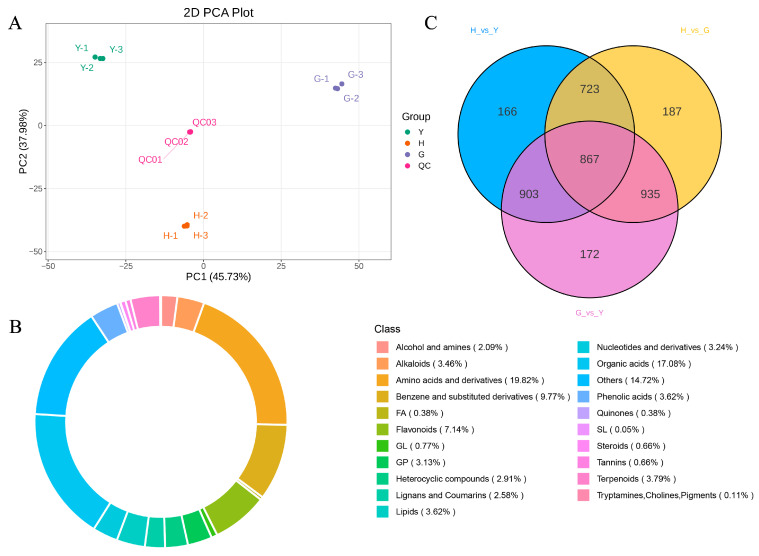
Metabolomic Analysis of *Lonicera macranthoides* Leaves, Flowers, and Fruits. (**A**) PCA results for the three tissues. (**B**) Metabolite classification, with different colors representing distinct metabolite categories. (**C**) Venn diagram of differentially accumulated metabolites across the three tissues.

This study further employed non-targeted metabolomics to analyze metabolites in flowers, leaves, and fruits of *Lonicera macranthoides*. Results showed that the three replicates of each sample and the QC sample clustered together, indicating that the sequencing data can be utilized for subsequent research (Figure 5A). Following LC-MS/MS analysis, metabolite identification was conducted by integrating data from self-constructed databases, public repositories, and in silico predicted databases, complemented by the metDNA annotation method. This integrated approach yielded a total of 4961 reliably identified metabolites (Table S8). The top three classes by abundance were Amino acids and derivatives (19.82%), Organic acids (17.08%), and Benzene and substituted derivatives (9.77%) (Figure 5B). Differentially accumulated metabolites (DAMs) screening identified 867 DAMs across all samples (Figure 5C). Volcano plots further visualized DAMs among individual samples (Figure S3). Among G vs. Y DAMs, 1030 were upregulated and 1537 were downregulated (Figure S3A). In H vs. G DAMs, 1231 were upregulated and 1114 were downregulated (Figure S3B). In H vs. Y DAMs, 967 were upregulated and 1383 were downregulated (Figure S3C). Cluster analysis of DAMs revealed that up- and downregulated DAMs clustered together (Figure S4).

**Figure 6:**
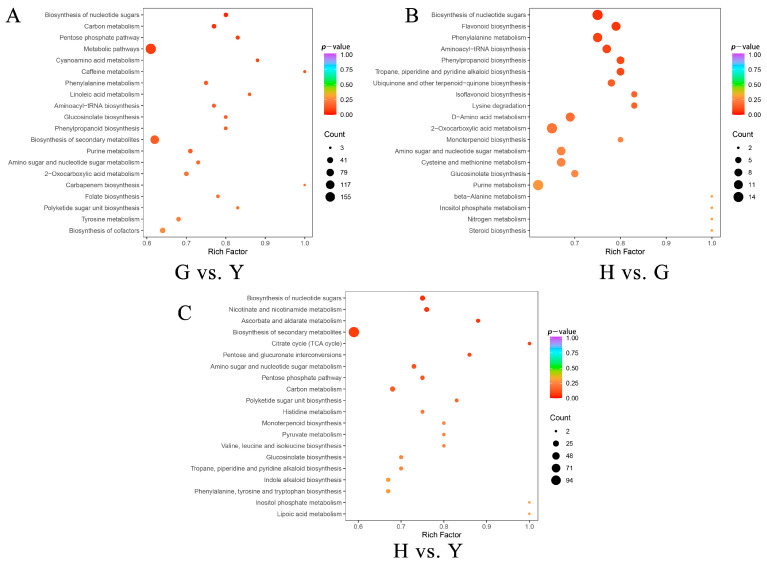
KEGG pathway enrichment analysis of differentially accumulated compounds. (**A**) G vs. Y. (**B**) H vs. G. (**C**) H vs. Y.

The DAMs were further mapped to KEGG pathways, and subsequent enrichment analysis revealed that metabolic pathways and biosynthesis of secondary metabolites accounted for a relatively high proportion among all significantly enriched pathways in pairwise comparisons across the three tissues of *Lonicera macranthoides* (Figure S5). Additionally, we analyzed the top 20 DAMs sorted by minimum *p*-value (Figure 6). Results indicate that in G vs. Y, the most significant pathways were Biosynthesis of nucleotide sugars and Carbon metabolism (Figure 6A). In G vs. Y, the most significant pathway was Biosynthesis of nucleotide sugars (Figure 6B). In H vs. Y, the most significant pathways were Biosynthesis of nucleotide sugars and Nicotinate and nicotinamide metabolism (Figure 6C).

### 3.5. Integrated Analysis of Metabolomics and Transcriptomics

**Figure 7:**
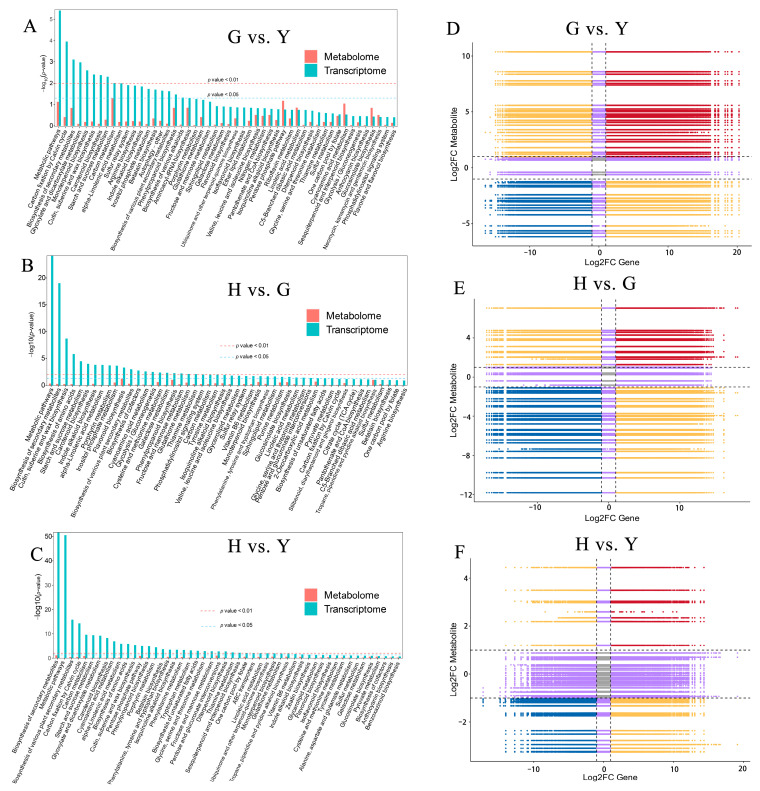
Figure 6 Conjoint analysis of DEGs in the transcriptome and DAMs in the metabolome. (**A**–**C**) The metabolic pathways commonly enriched in both the transcriptome and metabolome. (**D**–**F**) Nine-quadrant plot with the horizontal axis representing Log_2_FC of DEGs and the vertical axis representing Log_2_FC of DAMs.

To further investigate the association between DEGs and DAMs, this study conducted a conjoint analysis of metabolomics and transcriptomics. In the G vs. Y, G vs. Y, and H vs. Y comparisons, the top 50 shared pathways showed the highest enrichment in Metabolic pathways and Biosynthesis of secondary metabolites (Figure 7A–C). Additionally, enrichment in Cutin, suberine and wax biosynthesis, Starch and sucrose metabolism, and Carbon fixation by Calvin cycle was broadly observed. Using quantitative values of genes and metabolites across all samples, correlation analysis was performed. Within each differential group, correlations meeting the criteria of a Pearson correlation coefficient absolute value > 0.8 and *p*-value < 0.05 were visualized (Figure 7D–F). Results indicated that in the three-group comparisons, the correlations in the third and seventh quadrants reflected positive correlations between genes and metabolites, implying that changes in metabolite accumulation may be driven by gene positive regulation. Correlations in the first and ninth quadrants reveal opposite differential expression patterns between genes and metabolites, suggesting that changes in metabolite expression may result from gene negative regulation. Correlation clustering analysis based on DEGs and DAMs correlation results revealed that comparisons G vs. Y, G vs. Y, and H vs. Y simultaneously contained both positive and negative correlations involving DEGs and DAMs (Figure S6).

### 3.6. Tissue-Specific Differences in the Biosynthesis of Hederagenin-Based Saponins

**Figure 8:**
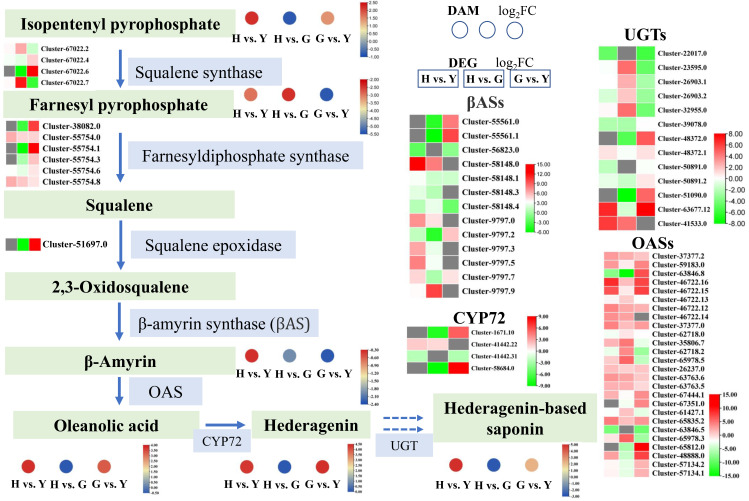
DEGs and DAMs in the biosynthesis pathway of hederagenin-based saponins. Green-background boxes indicate intermediate or final metabolites, while green-background boxes denote enzymes encoded by genes. Circles represent DAM comparisons within the metabolome, and rectangles indicate DEG comparisons within the transcriptome.

Hedera-based saponins represent one of the most critical metabolites in *Lonicera macranthoides*. To investigate their differential accumulation in leaves, flowers, and fruits, we analyzed metabolites and gene expression within their synthetic pathway (Figure 8). In the hedera-based saponin biosynthesis pathway, genes encoding squalene synthase, farnesyldiphosphate synthase, squalene epoxidase, β-amyrin synthase (βAS), and oleanolic acid synthase (OAS) showed significantly up-regulated expression in flowers and fruits. Similarly, the accumulation levels of isopentenyl pyrophosphate, β-amyrin, oleanolic acid, hederagenin, and hederagenin-based saponins were significantly higher in flowers or fruits than in leaves. Notably, farnesyl pyrophosphate significantly accumulated in leaves, suggesting potential transport from leaves to flowers or fruits. Furthermore, genes involved in the biosynthesis of hedera-based saponins, such as *CYP72* and *UGT*, were also significantly expressed in flowers or fruits (Figure 3). These results indicated that flowers and fruits of *Lonicera macranthoides* served as important synthetic tissues for hedera-based saponins.

### 3.7. Tissue Differences in Flavonoid Biosynthesis

Flavonoids exhibit prominent antioxidant activities, and *Lonicera macranthoides* is enriched in these bioactive compounds. To further elucidate differences in flavonoid metabolite accumulation among leaves, flowers, and fruits of *Lonicera macranthoides*, an integrated metabolome and transcriptome analysis was performed on these three distinct tissues. In the fruits versus leaves (G vs. Y) comparison, numerous genes involved in the flavonoid biosynthesis pathway were upregulated, accompanied by significant accumulation of several corresponding metabolites (Figure S7). These findings collectively indicate that flavonoid biosynthesis is substantially more active in fruits than in leaves. Conversely, in the flowers versus fruits (H vs. G) comparison, numerous flavonoid biosynthesis-related genes were downregulated, and several metabolites also showed significant downregulation, indicating substantially higher flavonoid accumulation in fruits than in flowers (Figure S8). In the flowers vs. leaves comparison, genes in the flavonoid biosynthesis pathway showed both significant upregulation and downregulation, with only a few metabolites exhibiting significant downregulation (Figure S9). These results indicated that the fruits of *Lonicera macranthoides* were likely the primary organ for flavonoid biosynthesis.

## 4. Discussion

*Lonicera macranthoides* is a valuable traditional Chinese medicinal plant whose bioactive compounds effectively treat various diseases [1]. The whole plant contains organic compounds such as chlorogenic acid [4], lonicerin [21], loniceroside A [22], and hederagenin [1], most of which exhibit significant biological activity in vitro or in vivo. The flowers accumulate secondary metabolites including macranthoidin, dipsacoside, macranthoside, saponin, and lonijaposide [23,24,25]. The leaves contain compounds such as flavonoids and iridoids [26,27]. *Lonicera caerulea* is a species within the honeysuckle family, whose fruits contain significant amounts of active compounds such as phenolic compounds, flavonoids, and anthocyanins [28]. Previous studies have analyzed metabolite biosynthesis in the flower buds, stems, and leaves of *Lonicera macranthoides* through metabolomics and transcriptomics, providing a foundation for understanding the regulatory mechanisms governing the accumulation of flavonoid compounds in honeysuckle [29]. This study compared differences in the accumulation of saponins and flavonoid metabolites among blooming flowers, ripe leaves, and ripe fruits through analysis of their transcriptomes and metabolomes. *Lonicera macranthoides* and *Lonicera japonica* are two widely cultivated plants in the genus Lonicera. Comparative analysis of their metabolomes, transcriptomes, and genomes revealed that *Lonicera macranthoides* contains 2000-fold higher levels of hederagenin-based saponins than *Lonicera japonica*, with significantly higher expression of genes involved in synthesizing these saponins [3]. Among the three tissues selected for this study, the flowers and fruits were found to be the primary tissues for saponin synthesis in *Lonicera macranthoides*, while the fruits were identified as the main site for flavonoid synthesis.

Flavonoid metabolites provide robust support for life maintenance, stress adaptation, growth, and development. As a key features of terrestrial plants, flavonoid s biosynthetic pathways exhibit diversity across plant species [30]. The biosynthesis of flavonoids proceeds through the phenylpropanoid metabolic pathway, which encompasses the biosynthesis of isoflavones, flavones, flavonols, anthocyanins, and proanthocyanidins [31]. Through transcriptomic and metabolomic analyses, genes such as *CHS*, *FLS*, and *F3’H* were found to exhibit significant differential expression in different tissues of *Rhododendron pulchrum* [32]. Transcriptomic sequencing and metabolomic studies revealed 105 differentially accumulated flavonoids (DAFs) and 1858 DEGs in goji berries from three distinct regions, with genes such as *CHI*, *CHS*, and *FLS* regulating flavonoid accumulation [33]. In current research, *CHS*, *CHI*, *FLS*, and other enzymes showed significantly upregulated expression in the fruits of *Lonicera macranthoides*, suggesting that its fruits may serve as the primary organ for flavonoid accumulation. *Lonicera caerulea* is a closely related species to *Lonicera macranthoides*, and its fruits similarly contain abundant flavonoid metabolites [28]. CHS serves as the first rate-limiting enzyme in the flavonoid biosynthetic pathway. Fourteen CHS family members have been identified in bitter buckwheat (*Fagopyrum tataricum*), among which *FtCHS3*, *FtCHS4*, *FtCHS5*, and *FtCHS6* exhibit higher transcriptional levels in floral tissues [34]. *CHS*-, *CHI*-, and *CHIL*-deficient rice mutants all lack extractable flavonoids, supporting the critical roles of CHS and CHI in flavonoid biosynthesis [35]. Overexpression of the chrysanthemum *CmFNS* and *CmFLS* genes in tobacco showed accumulation of flavonoids and flavonols, but less anthocyanins. In five flower color varieties with different anthocyanin levels, the expression of *CmFLS* and *CmFNS* showed opposite trends, indicating that *CmFNS* and *CmFLS* are important regulators of flavonoid and flavonol biosynthesis, respectively [36]. Previous studies have revealed that flavonoid content is higher in leaves than in stems and flower buds [29]. In this study, flavonoids were found to accumulate primarily in ripe fruits, followed by leaves. These findings indicate that fruits and leaves represent potential raw materials for extracting flavonoid metabolites.

In *Lonicera macranthoides*, hederagenin based saponins include macranthoidin B, dipsacoside B, and macranthoidin A, which belong to triterpenoid saponins [1,3,37]. The biosynthesis of hederagenin-based saponins includes the mevalonate (MVA) and the methylerythritol phosphate (MEP) pathway; in the formation and modification of the triterpene skeleton, squalene cyclooxygenase, β-Amyrin synthase, oleanolic acid synthas, CYPs, and UGTs are very critical enzymes [6,7,38,39]. In present study, at least 72 *CYP* genes and 12 *UGT* genes were identified, exhibiting differential expression in leaves, flowers, and fruits of *Lonicera macranthoides*, suggesting that Hederagenin-based saponins may be synthesized in all three tissues. Consistent with previous studies, this research identified four *CYP72A* family genes in the transcriptome results that may be involved in saponin synthesis. Additionally, the presence of a *UGT73P1* homolog, Cluster-41533.0, suggests it may be a key gene in the saponin synthesis pathway [3]. Furthermore, compared to leaves, *OAS* and *βAS* were significantly up-regulated in flowers and fruits, indicating that flowers and fruits are the primary tissues for hederagenin-based saponin biosynthesis. *LmOAS1*, cloned from *Lonicera macranthoides*, exhibits efficient catalytic activity for oleanolic acid production from β-amyrin when expressed in yeast, with mutations K145E, P146S, and A148S impairing its enzymatic activity [3]. *OAS1* is a type II cytochrome oxidase homologous to the *CYP716A* subfamily genes, primarily functioning to catalyze the oxidation of β-amyrin to oleanolic acid in plants [40]. In the current study, 26 *OAS* homologs were identified, which showed differential expression in flowers and fruits. Five CYPs in the *CYP72A* subfamily of *barbarea vulgaris* may be associated with saponin biosynthesis, and these genes were co-localized, while another gene associated with saponin biosynthesis, *CYP716As*, have no QTL localization [41]. Transcriptome sequencing of *Kalopanax septemlobus* identified at least 110 CYPs. Specifically, *CYP716A94* encodes β-amyrin 28-oxidase, which catalyzes the conversion of β-amyrin to oleanolic acid, whereas *CYP72A397* encodes oleanolic acid 23-hydroxylase that further converts oleanolic acid to hederagenin [42]. Six UGTs of the UGT73C subfamily in *Barbarea vulgaris* are able to glycosylate saponins and monosaccharides at positions 3 and/or 28, and some UGTs can efficiently form disaccharide saponins [43]. Previous studies have demonstrated that LmMYB15 is involved in regulating the metabolic processes of chlorogenic acid and phenylpropanoids in *Lonicera macranthoides* [44]. In the current transcriptomic data, a large number of transcription factors were found to be differentially expressed, and these transcription factors may play crucial roles in metabolite synthesis across different tissues (Figure S2). Current research has elucidated the differential accumulation of metabolites in the flowers, leaves, and fruits of *Lonicera macranthoides* at the transcriptomic and metabolomic levels. However, the biochemical characteristics of these tissues remain unexplored, which will be a focus for future studies. Furthermore, the regulatory mechanisms underlying key differential metabolites require further investigation, and it is anticipated that these questions will be resolved in the future.

## 5. Conclusions

In the present study, transcriptomic and metabolomic analyses were performed on leaves, flowers, and fruits of *Lonicera macranthoides*. Transcriptome sequencing identified 42,811 DEGs between fruits and leaves, among which 21,387 genes were significantly upregulated and 21,424 were downregulated. Comparing flowers and fruits yielded 40,679 DEGs, with 19,907 upregulated and 20,772 downregulated. Comparing flowers and leaves revealed 27,794 DEGs, including 13,582 upregulated and 14,212 downregulated. A total of 7321 DEGs were found to be commonly expressed across flowers, fruits, and leaves. Among the top 20 KEGG pathways enriched by these DEGs, metabolic pathways, biosynthesis of secondary metabolites, and starch and sugar metabolism pathways were prominently represented. Among CYP and UGT enzymes involved in hederagenin-based saponin biosynthesis, at least 70 differentially expressed *CYP* genes were identified, with a higher number of these genes being upregulated in flowers or fruits. A total of 12 differentially expressed *UGTs* were detected, with 7 upregulated in fruits and 5 upregulated in leaves and flowers. Genes involved in flavonoid biosynthesis, including *CHI*, *CHS*, and *FLS*, exhibited differential expression predominantly in fruit. Metabolomics profiling identified 4961 metabolites. Among these metabolites, the most predominant classes were amino acids and their derivatives (19.82%), organic acids (17.08%), and benzene and substituted derivatives. A total of 867 DAMs were identified among the three tissue samples. A comparison between fruit and leaves revealed 1030 up-regulated and 1537 down-regulated DAMs. Between flowers and fruit, 1231 DAMs were up-regulated and 1114 were down-regulated, whereas between flowers and leaves, 967 DAMs were up-regulated and 1383 were down-regulated. KEGG pathway classification of DAMs revealed a high proportion of metabolic pathways and secondary metabolite biosynthesis. Conjoint analysis of transcriptomic and metabolomic data revealed that among the top 50 shared pathways, “Metabolic pathways” and “Biosynthesis of secondary metabolites” exhibited the highest enrichment. The DEGs and DAMs related to hederagenin-based saponin biosynthesis were significantly upregulated in both flowers and fruits, whereas those associated with flavonoid biosynthesis pathways showed significant upregulation specifically in fruits. In conclusion, the present study reveals the differential accumulation of key metabolites across distinct tissues of *Lonicera macranthoides*, providing omics-based insights into the efficient extraction and utilization of metabolites from different tissues of this species. Furthermore, this work lays a foundation for subsequent investigations into the molecular regulatory mechanisms underlying the biosynthesis of key metabolites.

## Data Availability

The raw RNA-seq data have been uploaded to the Sequence Read Archive (SRA) database. The accession number is PRJNA1335771. All supporting data is included in the Supplementary Materials. For further inquiries, please contact the first author or corresponding author.

## Supplementary Materials

The following supporting information can be downloaded at: https://www.mdpi.com/article/10.3390/metabo16010005/s1, Figure S1. GO functional enrichment of DEGs; Figure S2. Identification of transcription factors in differentially expressed genes; Figure S3. Volcano plot of DEGs; Figure S4. Cluster Analysis of DAMs; Figure S5. KEGG metabolic pathway enrichment analysis of DAMs; Figure S6. Correlation Cluster Analysis of DEGs and DAMs; Figure S7. Comparing DEGs and DAMs in the flavonoid biosynthesis pathway between fruits and leaves; Figure S8. Comparing DEGs and DAMs in the flavonoid biosynthesis pathway between flowers and fruits; Figure S9. Comparing DEGs and DAMs in the flavonoid biosynthesis pathway between flowers and leaves; Table S1. Primers of genes used for RT-qPCR; Table S2. T3 Chromatography column mobile phase gradient conditions; Table S3. AB TripleTOF 6600 Mass Spectrometry conditions; Table S4. Statistical output of RNA-seq data for leaves, flowers, and fruits of *Lonicera macranthoides*; Table S5. RNA-seq assembly results for leaves, flowers, and fruits of *Lonicera macranthoides*; Table S6. Functional annotation of genes from RNA-seq data of leaves, flowers, and fruits of *Lonicera macranthoides*; Table S7. Statistics of DEGs in *Lonicera macranthoides* leaves, flowers, and fruits based on RNA-seq analysis; Table S8. Metabolite ID, integral value, and corresponding name detected in *Lonicera macranthoides* flowers, leaves, and fruits.
