# Supplementary figures and images for: Transcriptome and Metabolome Analyses Reveal Differences in the Accumulation of Key Metabolites in Various Tissues of Lonicera macranthoides
Source: Metabolites. 2025 Dec 22;16(1):5. doi: 10.3390/metabo16010005 (PMC12843617; doi:10.3390/metabo16010005)

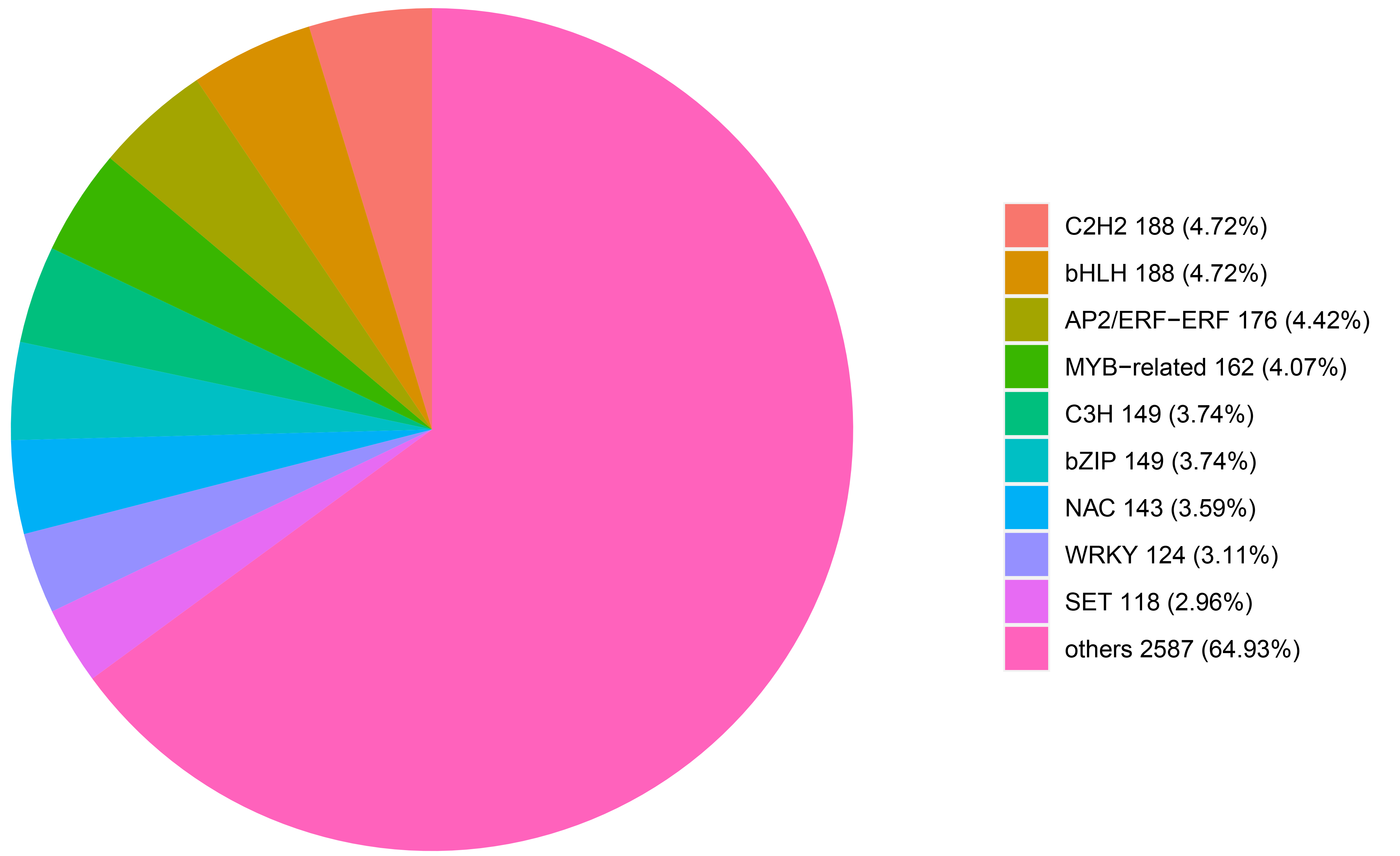

Supplement: Supplementary file 1 [file metabolites-16-00005-s001.zip › Figure S2.tif]

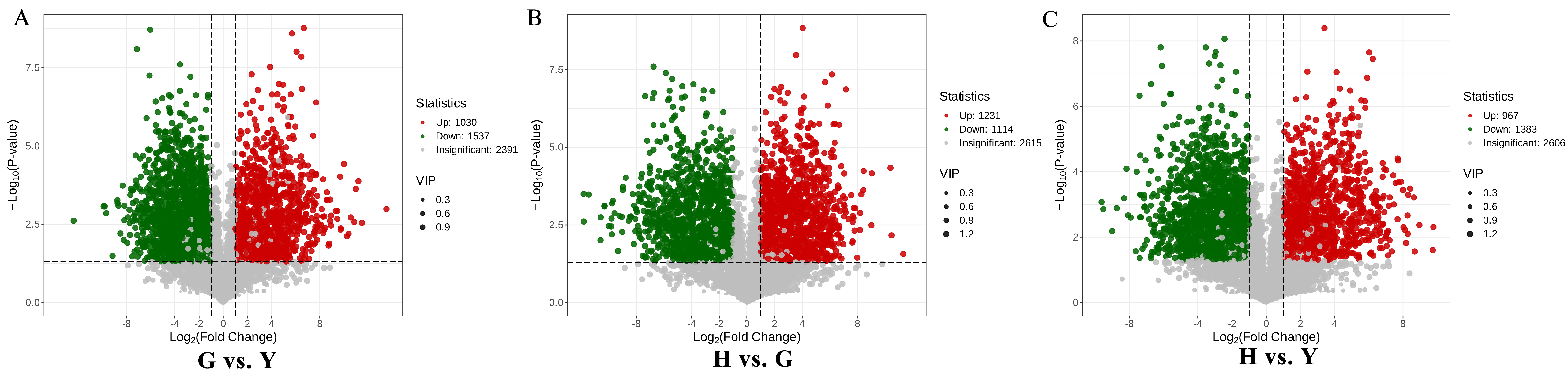

Supplement: Supplementary file 1 [file metabolites-16-00005-s001.zip › Figure S3.tif]

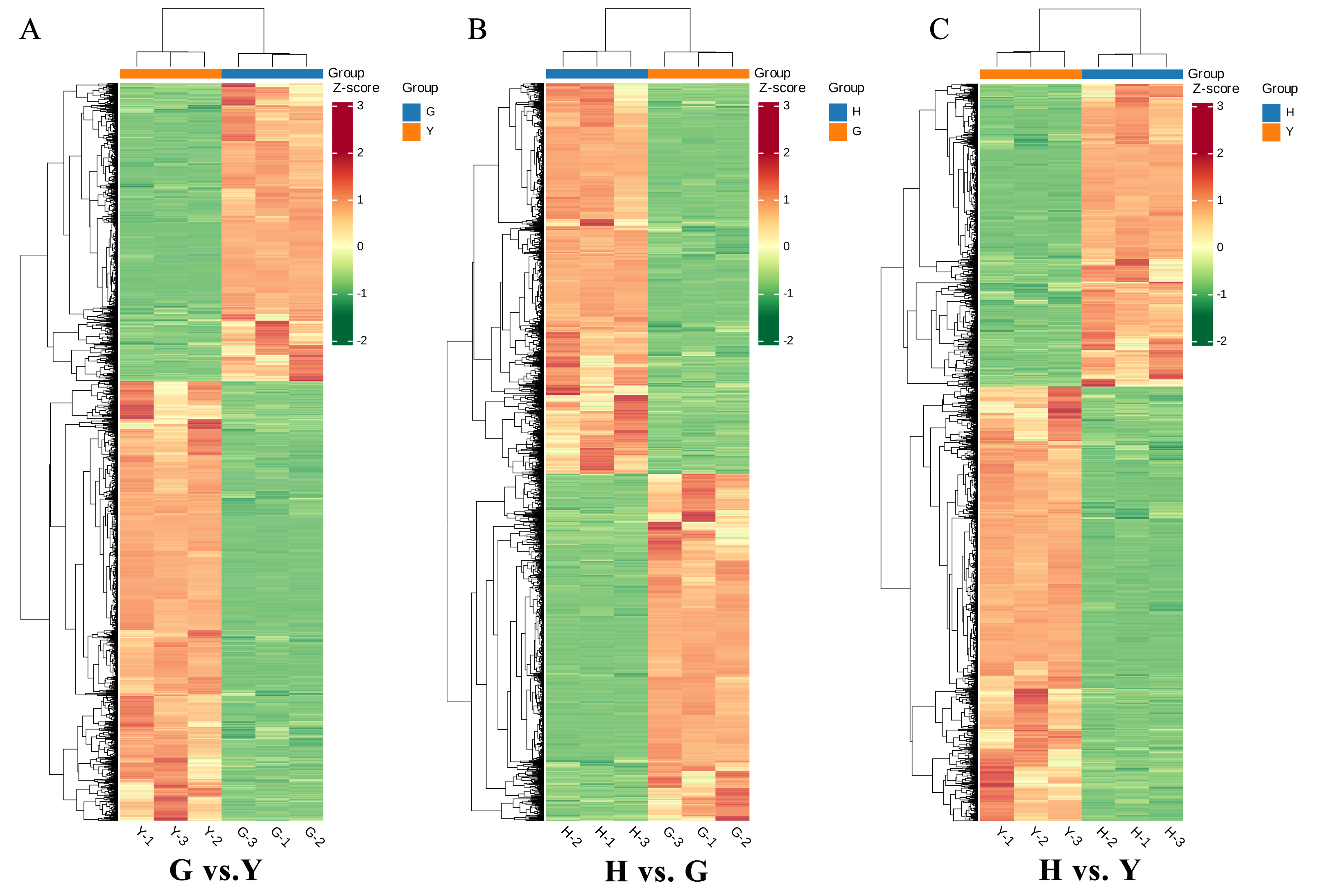

Supplement: Supplementary file 1 [file metabolites-16-00005-s001.zip › Figure S4.tif]

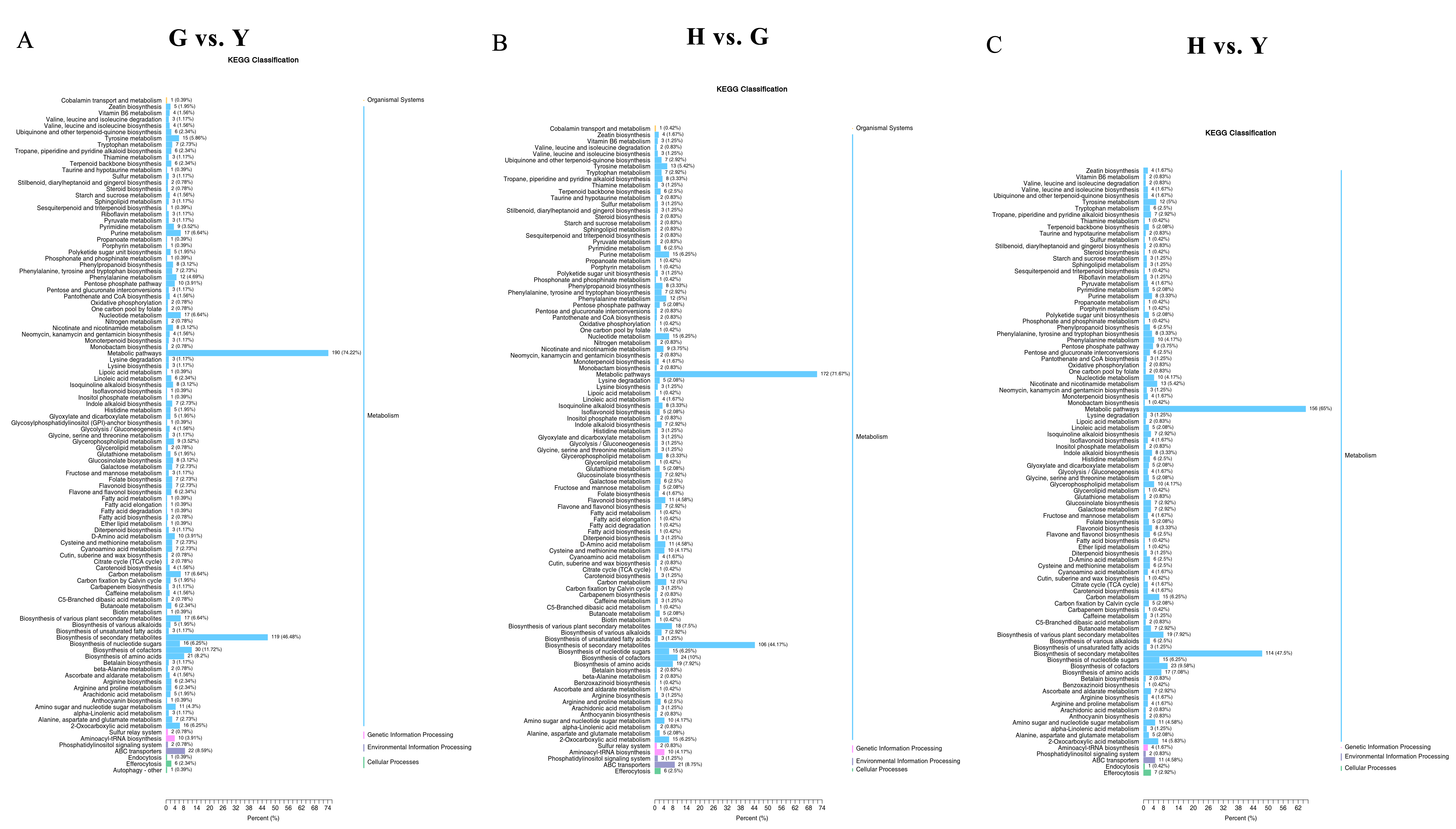

Supplement: Supplementary file 1 [file metabolites-16-00005-s001.zip › Figure S5.tif]

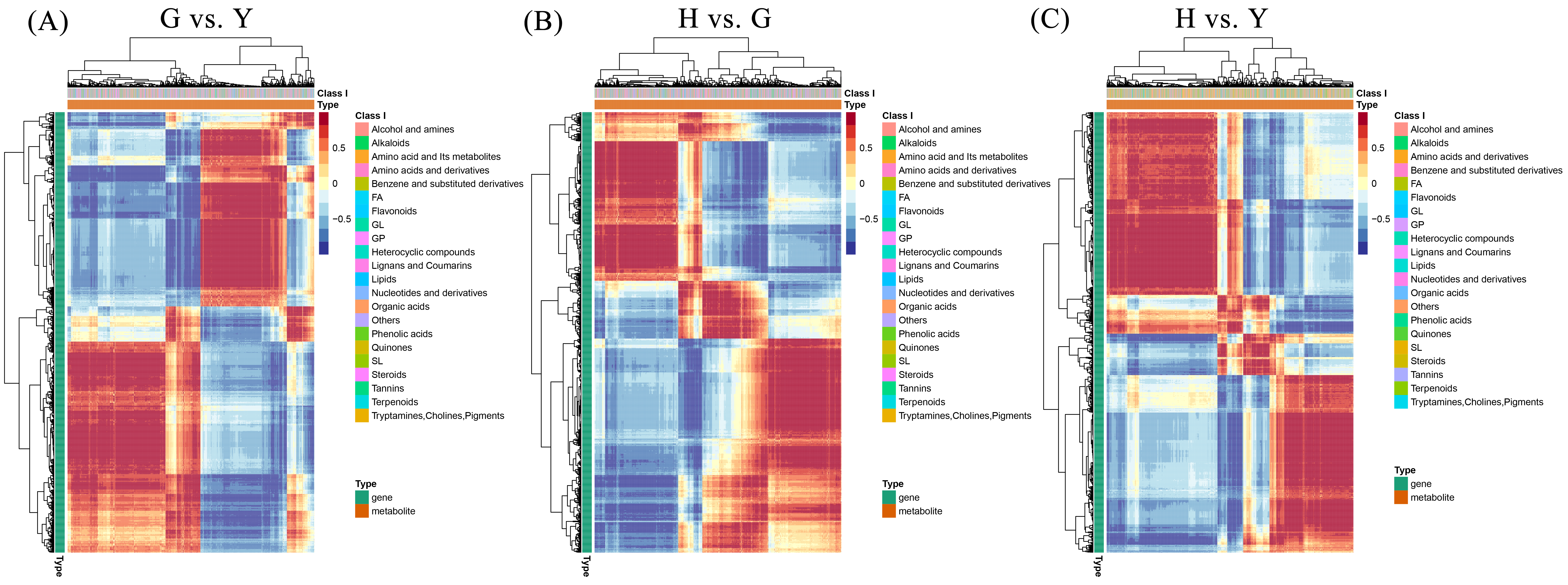

Supplement: Supplementary file 1 [file metabolites-16-00005-s001.zip › Figure S6.tif]

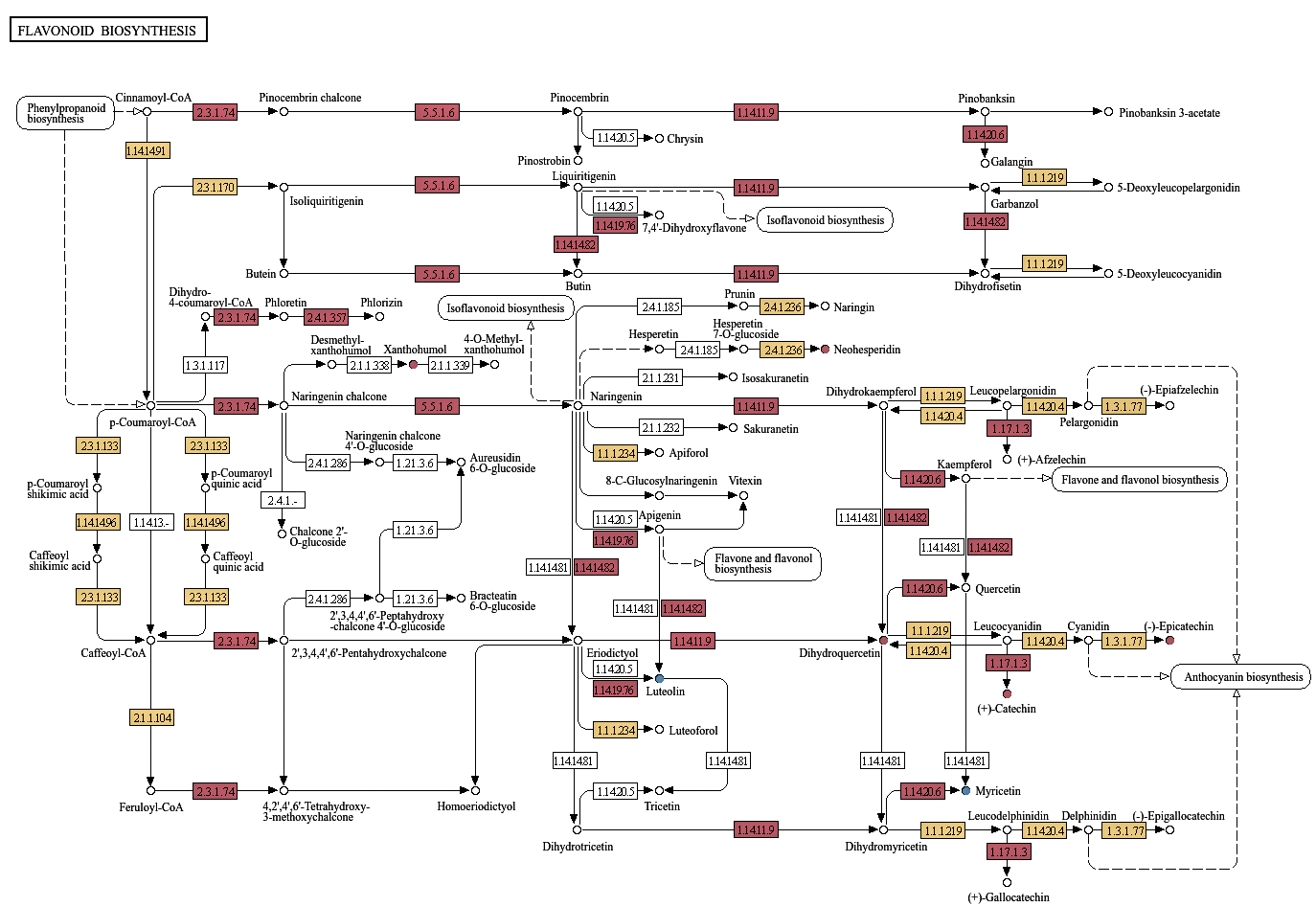

Supplement: Supplementary file 1 [file metabolites-16-00005-s001.zip › Figure S7.tif]

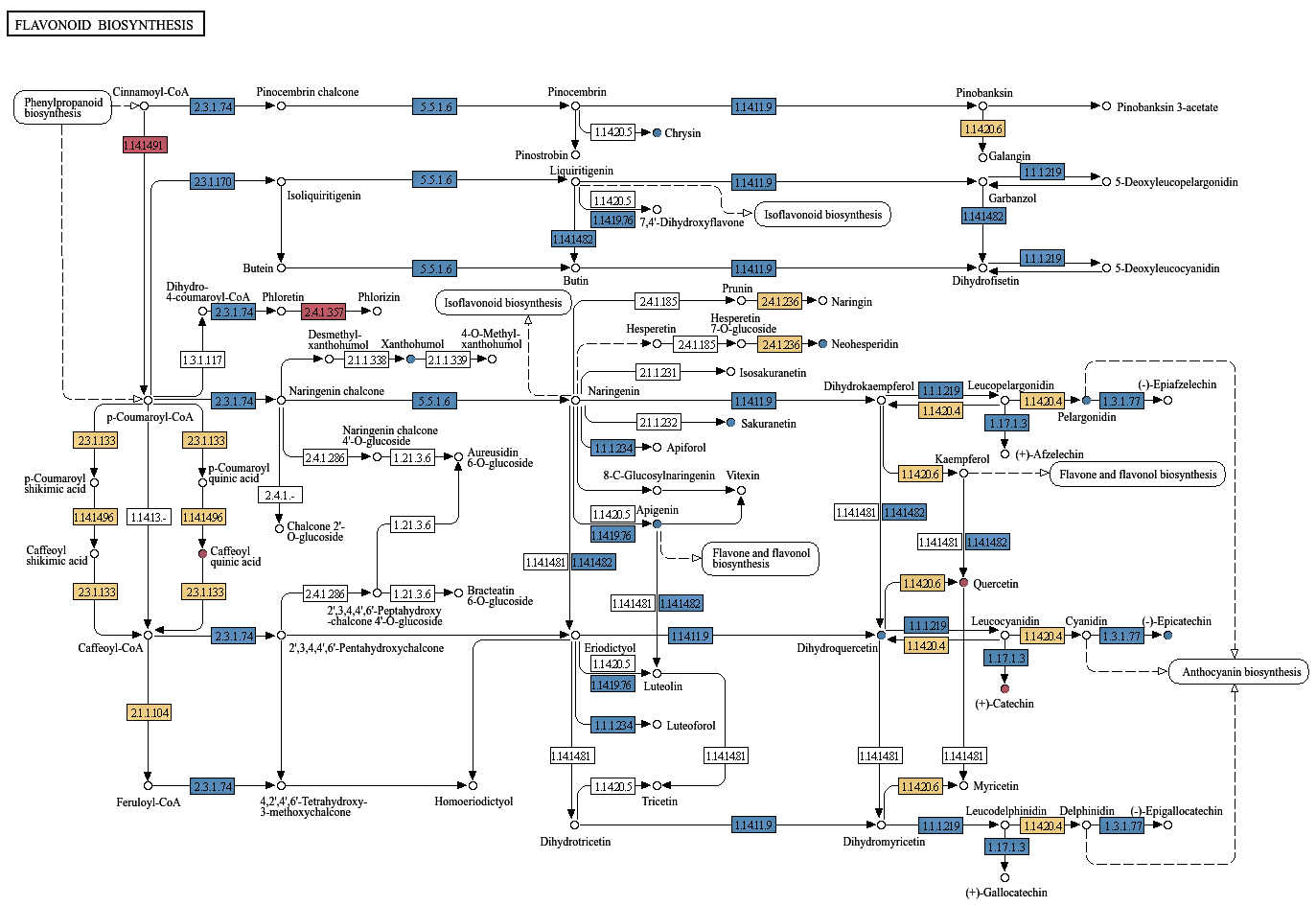

Supplement: Supplementary file 1 [file metabolites-16-00005-s001.zip › Figure S8.tif]

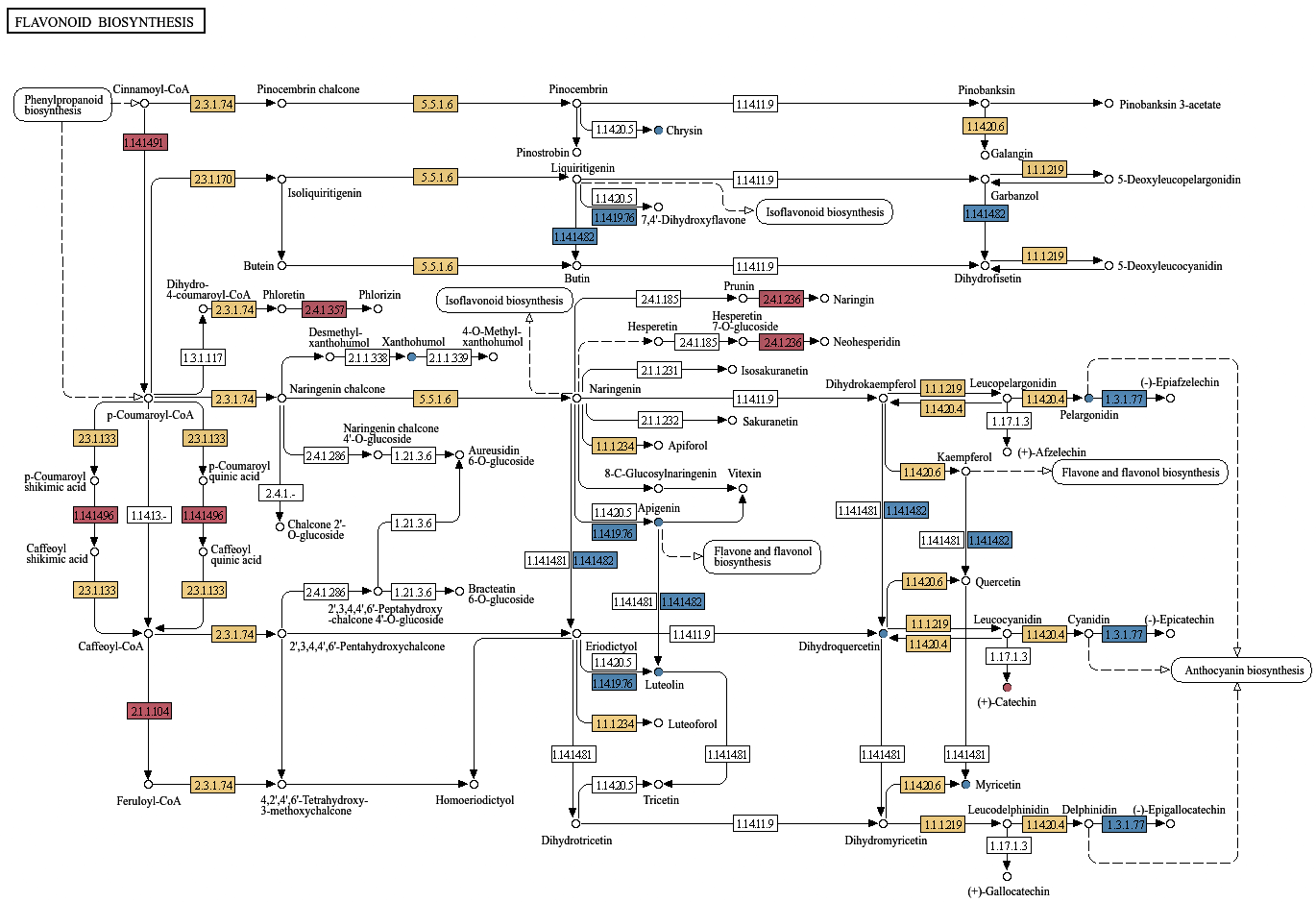

Supplement: Supplementary file 1 [file metabolites-16-00005-s001.zip › Figure S9.tif]
